# US-Guided Interventional Procedures for Total Hip Arthroplasty

**DOI:** 10.3390/jcm13133976

**Published:** 2024-07-08

**Authors:** Domenico Albano, Roberto Cintioli, Carmelo Messina, Francesca Serpi, Salvatore Gitto, Laura Mascitti, Giacomo Vignati, Pierluigi Glielmo, Paolo Vitali, Luigi Zagra, Žiga Snoj, Luca Maria Sconfienza

**Affiliations:** 1 IRCCS Istituto Ortopedico Galeazzi, 20161 Milan, Italy; carmelomessina.md@gmail.com (C.M.); fr.serpi@gmail.com (F.S.); sal.gitto@gmail.com (S.G.); paolo.vitali@unimi.it (P.V.); prof@luigizagra.it (L.Z.); io@lucasconfienza.it (L.M.S.); 2 Dipartimento di Scienze Biomediche, Chirurgiche ed Odontoiatriche, Università degli Studi di Milano, 20122 Milan, Italy; 3 Postgraduate School of Diagnostic and Interventional Radiology, Università degli Studi di Milano, Via Festa del Perdono 7, 20122 Milan, Italy; cintioli.rc@gmail.com (R.C.); mascittilra@gmail.com (L.M.); giacomovignati95@gmail.com (G.V.); 4 Dipartimento di Scienze Biomediche per la Salute, Università degli Studi di Milano, 20122 Milan, Italy; 5 Clinical Radiology Institute, University Medical Centre Ljubljana, 1000 Ljubljana, Slovenia; ziga.snoj@gmail.com; 6 Department of Radiology, Faculty of Medicine, University of Ljubljana, 1000 Ljubljana, Slovenia

**Keywords:** hip, arthroplasty, ultrasound, guidance, intervention, aspiration, iliopsoas, trochanter, corticosteroid, lidocaine

## Abstract

In patients with total hip arthroplasty (THA) with recurrent pain, symptoms may be caused by several conditions involving not just the joint, but also the surrounding soft tissues including tendons, muscles, bursae, and peripheral nerves. US and US-guided interventional procedures are important tools in the diagnostic work-up of patients with painful THA given that it is possible to reach a prompt diagnosis both directly identifying the pathological changes of periprosthetic structures and indirectly evaluating the response and pain relief to local injection of anesthetics under US monitoring. Then, US guidance can be used for the aspiration of fluid from the joint or periarticular collections, or alternatively to follow the biopsy needle to collect samples for culture analysis in the suspicion of prosthetic joint infection. Furthermore, US-guided percutaneous interventions may be used to treat several conditions with well-established minimally invasive procedures that involve injections of corticosteroid, local anesthetics, and platelet-rich plasma or other autologous products. In this review, we will discuss the clinical and technical applications of US-guided percutaneous interventional procedures in painful THA that can be used in routine daily practice for diagnostic and therapeutic purposes.

## 1. Introduction

Hip osteoarthritis is a common condition worldwide with a huge impact on quality of life and morbidity [1]. Since the median age of death in the Western world is rising constantly, this often elderly related condition represents a major cause of pain and reduced mobility [2]. Along with the increased population age and prevalence of hip osteoarthritis, the replacement of the joint with total hip arthroplasty (THA) has been increasingly performed, now being considered the gold standard surgical treatment for this condition. Therefore, it represents one of the most common surgeries worldwide from the rural hospital to the tertiary orthopedic center [3].

## 2. Why Total Hip Arthroplasty Fails

THA is a safe surgical intervention, performed for more than a century with the first surgery made before 1900 [4]. Sometimes, quite uncommonly, THA fails with residual or recurrent hip pain due to several possible factors. Aseptic acetabular loosening is considered to be one of the most common causes of THA failure, but there are many others, including prosthetic joint infection (PJI), adverse local tissue reaction to prosthetic components, prosthetic rupture or dislocation, periprosthetic fracture, nerves injuries, muscles and tendons injuries or impingement, occurring days, months, and even years after surgery [5]. Patient-dependent factors such as drugs, smoking, and immune hypersensitivity to metal particles are some of the causes that contribute to the failure of a prosthetic implant. Environmental factors play a role as well, for instance, trauma, excessive mechanical stress, and impact with the metal components [6].

Hence, several causes should be considered and investigated in the diagnostic work-up of patients with painful THA. In this case, the correct diagnosis is essential to direct the patient to a correct and tailored treatment to alleviate the patient’s symptoms. In this setting, a multidisciplinary approach is needed with US-guided interventional procedures that may play an essential role in driving therapy either to identify the source of pain or to treat periprosthetic disorders [7,8].

## 3. US-Guided Procedures around the Hip

Interventional radiology involves the use of imaging as a guide for percutaneous procedures that, nowadays, are routinely performed for diagnostic and therapeutic purposes in musculoskeletal disorders [9]. US guidance is generally used since it is widely available, quick, radiation-free, and allows an accurate evaluation of soft tissues for establishing the diagnosis and for planning the trajectory of the procedure by avoiding neurovascular bundles under real-time monitoring of the needle [10]. Despite several structures around the hip being superficial and easily approached under US guidance, some procedures may be challenging, particularly in overweight patients with diffuse fat infiltration of periarticular muscles [11,12]. The most commonly injected medications are steroids, local anesthetics, and, less frequently, platelet-rich plasma (PRP) or other autologous products [10]. In this review, we will discuss the clinical and technical application of US-guided percutaneous interventional procedures in painful THA. We have not carried out a systematic review, but rather a narrative review, checking scientific papers written in English on this topic obtained from different databases (PubMed, Web of Science, SCOPUS) with no limitations on the year of publication.

### 3.1. Arthrocentesis and Periprosthetic Biopsy

Prosthetic joint infection (PJI) is one of the most common and important complications of total hip arthroplasty, with approximately one out of five cases of revision surgery being related to infection [13,14]. The diagnosis of PJI is not always easy given that blood tests (c-reactive protein, leukocytes, etc.) present limited reliability and that specific symptoms related to the infection (erythema, swelling, skin fistulae, and fever) are often not observed [15]. Therefore, a comprehensive evaluation is needed to rule out PJI, including clinical symptoms, history, and imaging scans such as X-ray, CT, MR, and labeled white blood cell bone scanning [7,16,17]. In this scenario, joint aspiration is very important and is recommended to retrieve synovial fluid that can be used for antimicrobial cultures and determining the dosage of inflammatory markers [14,18]. The identification of any infection and the responsible microorganism has a non-negligible impact on clinical management, since it may drive tailored antimicrobial therapy and the choice of one-stage or two-stage surgery, even if other elements must also be considered [19,20]. Hence, joint aspiration is helpful to complete the evaluation of painful THA, presenting an accuracy of approximately 65–70% [15]. Unfortunately, synovial fluid can be absent in up to half of patients with PJI [21,22,23]. In the case of “dry tap”, joint aspiration cannot be performed since no fluid returns to the syringe. In this scenario, sterile saline lavage of the joint, with saline injection and reaspiration for culture analysis, is a controversial option. Some authors consider saline lavage to have limited accuracy, and even sometimes harmful due to the risk of superimposed infection of the joint or contamination of the cultures by other microorganisms [24,25]. However, some others find it useful, despite there being no robust evidence [26]. An alternative option is US-guided periprosthetic synovium biopsy. This procedure seems to present lower sensitivity than joint aspiration (41% vs. 52%), but higher specificity (100% vs. 97%) and positive predictive value (100% vs. 92%) for the diagnosis of PJI in the pre-surgical setting of patients considered for revision surgery [27].

Sometimes, periprosthetic collections are not septic, but they are the result of inflammatory reactions to metallic components of prosthetic implants, leading to adverse reactions in local tissues—namely, metallosis and particle disease—with the development of pseudotumors that may affect bony and soft tissues [28]. Pseudotumors can be a mixture of solid and partially cystic tissue and require the revision of THA in 1–5% of patients [29]. US can show an anechoic fluid-filled mass, a solid hypo/hyperechoic mass with no significant fluid, or a mixed cystic/fluid-filled mass with thickened walls and ubiquitous solid components, with variable signal intensity on MRI [30]. Indeed, US has been proposed as a screening tool for adverse local tissue reaction after THA [31,32]. First, the anterior capsular distance, as measured with US in the anterior hip joint recess, is related to the size of the cup, but it does not seem to be related to the size of the femoral stem [33]. Then, the convex appearance of the iliofemoral ligament can be associated with intracapsular adverse local tissue reaction [34]. US-guided aspiration and biopsy can be performed to confirm the diagnosis, to exclude differential diagnosis including septic collections and soft tissue tumors with a similar imaging appearance (i.e., giant cell tumors), and to rule out a coexisting PJI [35].

#### Technical Considerations

**Figure 1:**
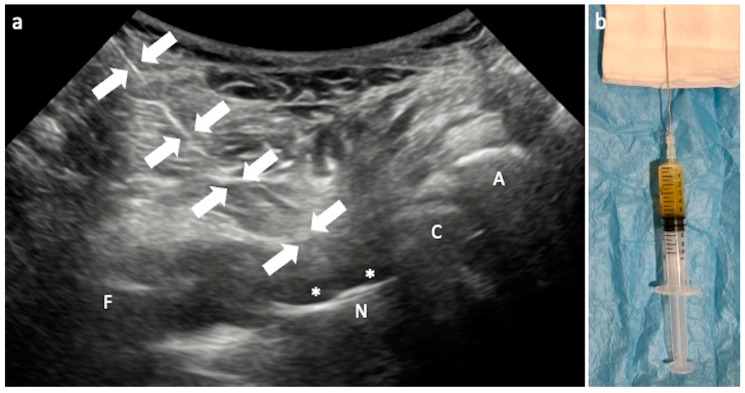
US-guided joint aspiration. (**a**) US image with convex probe on the long axis of the prosthetic neck and the needle (arrows) introduced with caudocranial approach to reach the prosthesis surrounded by effusion (asterisks); (**b**) six milliliters of synovial fluid has been collected in the syringe. F—femur; N—prosthetic neck; C—prosthetic cup; A—acetabulum.

Joint synovial fluid aspiration should be performed under US guidance with the patient supine and the probe oriented on the long axis of the prosthetic femoral neck, inserting the needle with in-plane from the caudal part of the transducer directly into the joint and advancing it until you touch the prosthesis. A 9–10 cm long spinal needle (20–22 Gauge) is generally used to aspirate effusion (Figure 1).

When the fluid is scarce, it might be helpful to proceed just medial or lateral to the prosthetic neck when trying to collect the small amount of effusion located posteriorly. In overweight patients, it would be better to approach the joint as closely as possible, inserting the needle with a more vertical orientation to shorten the trajectory of the needle itself. Of course, when septic collections are observed in periprosthetic superficial and deep soft tissues, drainage and laboratory analysis on aspirated fluid can be achieved in the same way.

**Figure 2:**
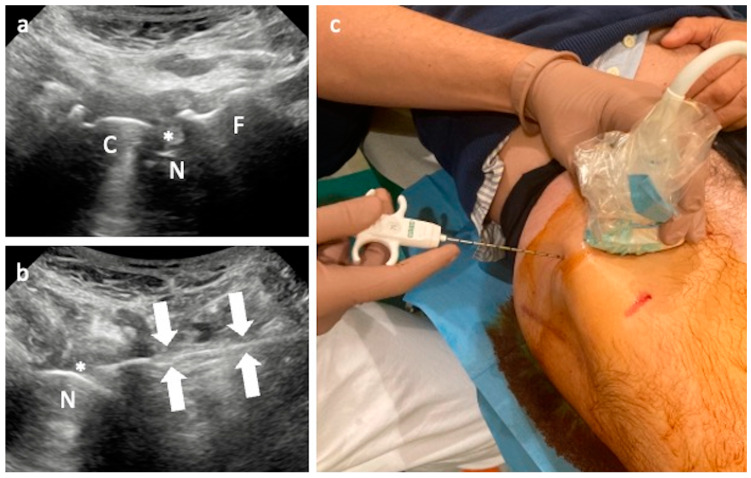
US-guided periprosthetic biopsy. Ultrasound in the long axis (**a**) and short axis (**b**) of the THA showing the hypoechoic synovium (asterisks) surrounding the prosthesis. The needle (arrows) is introduced from the lateral aspect of the hip (**c**), with the in-plane technique, with a perpendicular direction compared to the classical joint aspiration, advancing horizontally to be tangent to the prosthetic neck. N—prosthetic neck, F—femur; C—prosthetic cup.

When the fluid is absent, samples of periprosthetic tissue can be collected by using a Tru-Cut needle [36]. The internal stylet of the Tru-Cut can be manually advanced for 1–2 cm in the tiny periprosthetic tissue using a different entry point from the lateral side of the hip, at a variable depth based on patient habitus, passing above the greater trochanter, with the needle (10–15 cm length, 14–18 Gauge) being parallel to the floor and tangent to the prosthetic neck [27]. This procedure involves the use of local anesthesia injected into the skin/subcutaneous fat and periarticular tissues, while it is not required for joint aspiration (Figure 2). Then, the samples can be used for culture analysis, but in the absence of fluid, the leukocyte count, esterase, and percentages of neutrophils cannot be obtained.

### 3.2. Iliopsoas Tendinopathy

Iliopsoas tendinopathy due to impingement between the tendon itself and prosthesis components is an established cause of hip pain after THA [37] with an estimated incidence of up to approximately 30% of patients [38]. Mostly a prominence, oversizing or retroversion of acetabular component may induce friction, leading to tendinopathy and bursitis; alternatively, protruding screws or a large femoral head may determine this condition [38,39,40,41]. Iliopsoas tendinopathy in patients without THA is often related to internal extra-articular snapping syndrome, with a tendon “snap” heard during maneuvers of flexion, abduction, and external rotation of the hip, with a click sound produced when the Iliopsoas tendon snaps against the acetabulum when the hip of the patient returns to a neutral position [42]. After THA, iliopsoas impingement can be challenging to identify through clinical examination. Groin pain generally becomes worse during active and resisted flexion and active external rotation of the hip [43,44]. Imaging may drive the correct diagnosis and management, allowing the assessment of the position of prosthesis components, mainly using plain radiography and CT. Further, US and MRI can be used to detect tendinopathy, tendon tears, and bursitis, although the iliopsoas tendon may appear normal even in patients with impingement. A conservative approach with rest, painkillers, nonsteroidal anti-inflammatory drugs, physical therapy, and US-guided peri-tendinous injections of a mixture of local anesthetic and steroids is the first-line treatment [45]. Indeed, US-guided interventional procedures are helpful for both the diagnosis and treatment of iliopsoas bursitis and impingement syndrome. Indeed, a clinical response to local anesthetic injection is useful for confirming the iliopsoas tendon as the source of pain, making this “lidocaine test” part of the diagnostic work-up of patients with groin pain after THA. Further, the same procedure may be used for treating iliopsoas impingement, with corticosteroid and local anesthetic intra-bursal injection being proven to be effective in providing pain improvement in approximately 90% of patients [46]. When conservative treatment fails, the second step involves iliopsoas tenotomy and/or revision surgery [47].

#### Technical Considerations

**Figure 3:**
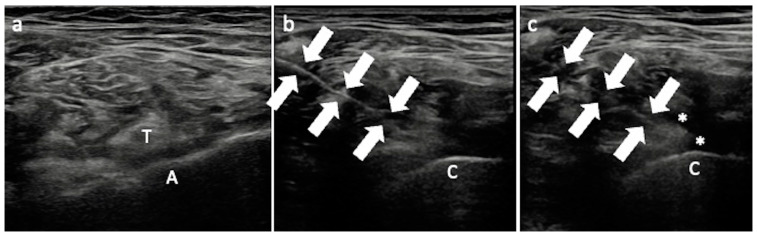
US-guided iliopsoas bursa injection. (**a**) The iliopsoas tendon (T) is scanned in the short axis at the acetabulum level; (**b**) the needle (arrows) is introduced laterally to reach the inferior part of the tendon; (**c**) once the target has been reached, the solution can be injected distending the iliopsoas bursa (asterisks). T—iliopsoas tendon; A—acetabulum; C—prosthetic cup.

The intervention is performed with the patient supine. A spinal needle is introduced in plane from the lateral aspect of the transducer, with the probe (generally a convex transducer is required) in the transverse position to scan the iliopsoas tendon in the short axis at the level of the acetabulum or the acetabular cup. The needle is advanced medially to reach the inferior part of the iliopsoas tendon, which is the target of the procedure (Figure 3).

Approximately 5 mL or less of local anesthetic—lidocaine 2% is routinely used at our institution—can be injected for the diagnostic test, and then any pain relief is evaluated in the minutes following the procedure. Regarding the therapeutic injection around the iliopsoas tendon, the approach is the same and includes the administration of a mixture of 1 mL of local anesthetic and 1 mL of corticosteroid—triamcinolone acetonide or metilprednisolone are used at our institution.

### 3.3. Greater Trochanteric Pain Syndrome

Gluteal tendon tendinopathy, tendon tears, and trochanteric bursitis are all features of so-called greater trochanteric pain syndrome (GTPS). This condition may determine lateral pain in patients with THA. Tendons are often injured during surgery or in the following months and years by altered biomechanics [48]. Gluteus medius tendon tears have higher significance than those affecting the gluteus minimus tendon [49]. In fact, the denervation of the gluteus minimus is often observed after implantation and its tendon may be released during surgery, minimizing the role of this tendon in GTPS after THA. US and MRI may show tendinopathy, partial or full-thickness gluteal tendon tears, calcifications, peritendinous edema, and bursitis [50]. GTPS is often managed conservatively with activity changes, nonsteroidal anti-inflammatory drugs, physical therapy, stretching and exercises for hip and thigh muscles, local injections of anesthetics and corticosteroids, and shock wave therapy [51,52]. These non-surgical alternatives have been proven to show good clinical results with a negligible rate of complications. Concerning local injections for GTPS, US guidance ensures higher effectiveness when compared with blinded injections [52]. US allows the direct visualization of the distended bursa, but, even in patients where there is no clear sign of bursitis, the injection of drugs is typically performed into the bursa providing pain reduction for the majority of patients with GTPS [53].

#### Technical Considerations

**Figure 4:**
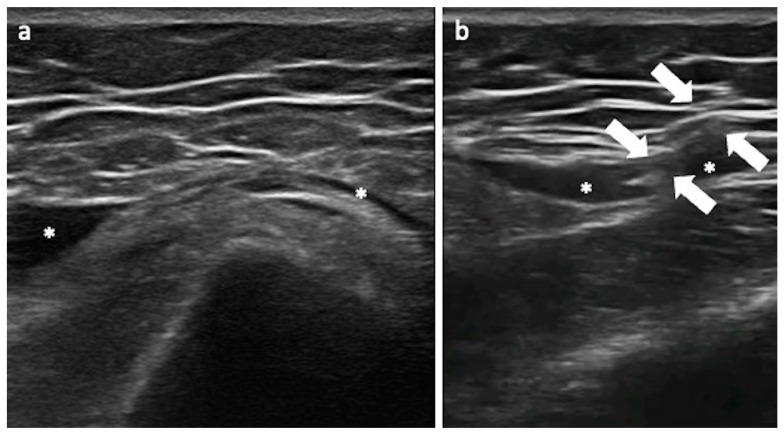
US-guided trochanteric injection. (**a**) US image shows the trochanteric bursa distended by effusion (asterisks); (**b**) when the bursa is distended, it is an easy target for the procedure, introducing the needle (arrows) with the in-plane approach into the bursa under US monitoring.

US-guided trochanteric injections require positioning the patient in lateral decubitus. Different approaches can be used: a linear or convex probe is positioned on the long axis of the gluteus medius tendon or in the short axis on the middle facet of the greater trochanter, and the needle (spinal 20–22 Gauge) is introduced and followed in plane from the caudal or lateral aspect of the transducer, respectively. The target is the trochanteric bursa or the peritendinous fat between the gluteus tendons/trochanteric facets and the gluteus maximus muscle/fascia latae (Figure 4) [54]. A solution of local anesthetic and corticosteroid is used for GTPS with good results. Alternative and valuable procedures include the US-guided needling of gluteal tendons and platelet-rich plasma injection without clear evidence supporting the use of these treatments over local corticosteroid injections [55,56]. Nevertheless, PRP might lead to higher pain relief in the long term compared to corticosteroid injections [57,58].

### 3.4. Nerve Injuries

Nerve injuries are rare but highly impactful complications of THA, with an incidence ranging from 0.6 to 4% [59]. Higher BMI is known as a negative prognostic factor [60]. Nerve injury can be caused due to stretching, direct tearing during hip replacement or after prosthesis dislocation, and compression related to bleeding, collection, and pseudotumors. The most commonly injured nerves are the sciatic, femoral, and lateral femoral cutaneous (LFCN). Clinical examination can raise diagnostic suspicion, with electromyography and other functional neurological studies being the front line in supporting clinicians, although they have no application in the acute phase. Imaging can be used to identify advanced signs of neuropathy, muscle denervation, and entrapment syndromes of the nerves, highlighting the causes of compression [61,62]. Injured nerves are generally thickened, hyperintense on fluid-sensitive sequences and hypoechoic on US, with a loss of perineural fat planes, and totally disrupted [63,64]. Nevertheless, in several cases, imaging does not allow the identification of pathological changes to small peripheral nerves. In this scenario, US-guided procedures may be used for both diagnostic and therapeutic purposes.

#### 3.4.1. Sciatic Nerve and Technical Considerations

An anatomical landmark for the sciatic nerve injury is the exit of the nerve out of the greater sciatic notch, where the sciatic nerve passes adjacent to the piriformis muscle [65,66]. Posterior/posterolateral access for THA surgery places the sciatic nerve at the highest risk of injury. Post-surgical hematoma is a cause of compression in the acute/subacute post-operative setting. In the case of neuropathy and severe pain after THA with clinical symptoms of complete dissection, or in patients with hematoma-driven impingement, surgery is the best choice, with urgent surgical exploration indicated for identifying and repairing the injury [65]. A delayed intervention carries lower success rates; therefore, correct timing is important. In the chronic setting where there is no surgical indication, percutaneous US-guided procedures may be less impactful solutions for symptom control [11]. Also, percutaneous injections in the perineural region often bring diagnostic information, with clinical response considered to be a positive test for neuropathy being the cause of pain. Hydrodissection is particularly indicated in case of sciatic nerve entrapment, injecting a large volume of saline mixed with lidocaine and corticosteroids to obtain the dissection of the nerve from the surrounding structures. Alternatively, Botulin A toxic intramuscular injection may be performed, since the induced atrophy of muscle fibers is thought to be useful for reducing volume and, therefore, mechanical stress on the sciatic nerve [12,67].

The patient is prone, and a linear or convex transducer is positioned in the short axis of the sciatic nerve between the biceps femoris and semimembranosus muscles located superficial to the nerve and the adductor magnus located below. If the injection must be performed proximally, the probe transducer can be moved cranial to the ischial tuberosity, where the nerve travels close to the ischiofemoral space. Then, a spinal needle should be introduced from lateral to medial with the in-plane technique to inject the solution around the nerve to debride the perisciatic fascial planes, obtaining the hydrodissection of the nerve.

#### 3.4.2. Lateral Femoral Cutaneous Nerve and Technical Considerations

The LFCN should be considered when THA is performed with anterior/anterolateral access. This nerve is prone to injury with this surgery approach given its superficial course. Indeed, it emerges deep into the inguinal ligament, providing branches that travel distally into the deep layer of subcutaneous fat and providing sensory innervation to the skin of the antero-lateral thigh. LFCN is a pure sensory nerve; therefore, nerve injury does not involve functional limitation, but only sensory loss and neuropathic pain with numbness, paresthesia, or even dysesthesia analogous to meralgia paresthetica [68,69]. Sensory deficits in a rather large number of patients decrease quality of life for months. LFCN injury is generally characterized by poor improvement in the first year after surgery [70], with good improvements after two years [71]. Ultrasound also provides value in the pre-operative setting for identifying and tracking LFCN and its anatomy. This may be useful for reducing the incidence of lesions in the operative setting [72,73]. Then, US-guided procedures may be useful for both diagnosis and treatment [74]. Indeed, anesthetic and corticosteroid injections provide pain relief and may be useful for confirming the diagnosis [12], with multiple injections during the nerve course improving the efficacy of the procedure [75].

**Figure 5:**
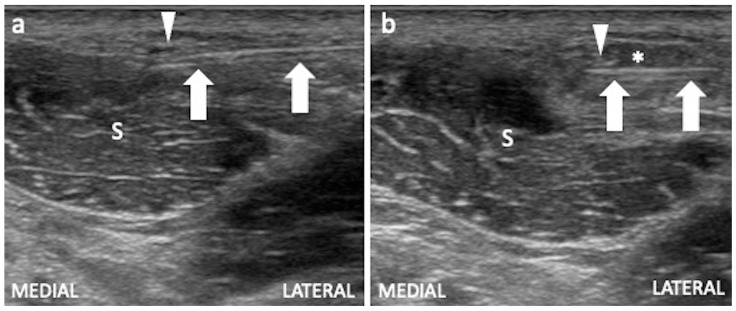
US-guided injection of the LFCN. (**a**) The LFCN (arrowhead) is identified in the short axis superficial to the sartorius muscle (S), sliding the probe distal to the anterior superior iliac spine and the needle (arrows) is introduced with an in-plane lateral to medial approach, placing the tip just below the nerve. (**b**) The mixture of local anesthetic and corticosteroid can be injected, monitoring the spread of the solution (asterisk) around the nerve.

For US-guided injection, the patient is supine, the linear transducer is in a transverse position to identify the LFCN immediately distal to the anterior superior iliac spine. The entry point is laterally advancing the tip of the needle with the in-plane technique up to the nerve medially. A solution of local anesthetics and steroids is administered around the nerve and its branches (Figure 5).

## 4. Perspectives

Most US machines are not specifically created for guiding interventional procedures. Nevertheless, some tools have been designed to improve needle visualization, and specific transducers/trackers are available. In this scenario, artificial intelligence could be helpful to improve the monitoring of needle advancement. In a previous study, the authors applied a convolutional neural network to increase the US visibility of the needle, allowing for the automatic identification of the needle insertion side, needle trajectory, and detection of the needle tip with almost 100% precision [76].

The improvement in fusion imaging systems to match real-time US with different imaging techniques (CT, MRI) has led to the introduction of this approach into clinical practice in several interventions (e.g., prostate biopsy) [77,78]. Some authors have investigated the use of this technology in musculoskeletal procedures, reporting interesting results [79,80,81]. Hence, despite US-guided interventional procedures around THA rarely being challenging, this technology may be useful in specific settings to improve the safety and efficacy of these interventions.

Virtual reality and augmented reality systems have been tested for use in training physicians and improving the safety and accuracy of interventional procedures [82,83,84,85,86]. This technology enables work to be carried out in a completely unreal setting (virtual reality) or just “augmenting reality” by overlaying digital content to the real operating room through electromagnetic or optical devices. These systems have been barely investigated for musculoskeletal interventional procedures, but it seems that they may improve the learning curve of trainees and make interventions more enjoyable and attractive, as well as improving the performance of real procedures performed on patients [87,88,89,90].

Another perspective for US in the treatment of musculoskeletal pain is high-intensity focused ultrasound technology [91]. It involves the use of ultrasound waves focused on a specific structure, leading to a local temperature increase, generally aimed at the ablation of biological tissue, specifically tumors [92,93,94,95,96,97,98,99]. Despite primary bone tumors and bone metastases being considered the preferred target in the musculoskeletal system, other possible applications have been proposed, including facet arthropathy and nerve ablations [100,101].

## 5. Conclusions

US and other imaging modalities are an essential part of the diagnostic work-up of failed THA to promptly identify the various complications that can be encountered after the prosthetic replacement of the hip. US-guided procedures can be applied to collect samples or to evaluate the response to local anesthetic injections to confirm or exclude diagnosis. Furthermore, US-guided interventional procedures are helpful, safe, and effective treatments for improving pain and function in patients with painful THA. These procedures are technically easy, but crucial to the correct management of these patients are an awareness of soft tissue and prosthesis anatomy, being familiar with THA complications, and having clear knowledge of the clinical indications of US-guided injections.

## Data Availability

No new data were created or analyzed in this study. Data sharing is not applicable to this article.
